# Two-Dimensional MXenes: Innovative Materials for Efficient Thermal Management and Safety Solutions

**DOI:** 10.34133/research.0542

**Published:** 2024-12-19

**Authors:** XiaoYan Hu, Qi Fan, Shengchao Wang, Yanxin Chen, Degao Wang, Ke Chen, Fangfang Ge, Wenhu Zhou, Kun Liang

**Affiliations:** ^1^ School of Materials Science and Chemical Engineering, Ningbo University, Ningbo, Zhejiang 315211, P. R. China.; ^2^ Zhejiang Key Laboratory of Data-Driven High-Safety Energy Materials and Applications, Ningbo Key Laboratory of Special Energy Materials and Chemistry, Ningbo Institute of Materials Technology and Engineering, Chinese Academy of Sciences, Ningbo 35201, P. R. China.; ^3^ University of Chinese Academy of Sciences, Beijing 100049, P. R. China.; ^4^ Advanced Interdisciplinary Sciences Research (AIR) Center, Ningbo Institute of Materials Technology and Engineering, Chinese Academy of Sciences, Ningbo 315201, P. R. China.; ^5^ Qianwan Institute of CNITECH, Ningbo 315336, P. R. China.; ^6^ Xiangya School of Pharmaceutical Sciences, Central South University, Changsha 410013, P. R. China.

## Abstract

MXenes, a class of 2-dimensional transition metal carbides and nitrides, have garnered important attention due to their remarkable electrical and thermal conductivity, high photothermal conversion efficiency, and multifunctionality. This review explores the potential of MXene materials in various thermal applications, including thermal energy storage, heat dissipation in electronic devices, and the mitigation of electromagnetic interference in wearable technologies. Recent advancements in MXene composites, such as MXene/bacterial cellulose aerogel films and MXene/polymer composites, have demonstrated enhanced performance in phase change thermal storage and electromagnetic interference shielding, underscoring their versatility and effectiveness. Although notable progress has been made, challenges remain, including the need for a deeper understanding of photothermal conversion mechanisms, improvements in mechanical properties, exploration of diverse MXene types, and the development of sustainable synthesis methods. This paper discusses these aspects and outlines future research directions, emphasizing the growing importance of MXenes in addressing energy efficiency, health, and safety concerns in modern applications.

## Introduction

Over the past decade, the utilization of thermal energy has garnered a lot of attention, leading to the advancement of related technologies. These technologies include solar energy harnessing [1], heat dissipation in electronic devices [2], thermal therapy [3], and fire prevention or flame retardation in batteries [4]. High thermal conductivity and efficient optical–thermal conversion are essential for maximizing thermal energy utilization. While various materials—including metals [5], inorganic nonmetals [6], semiconductors [7], and organic polymers [8]—have been widely employed, they often fail to meet the specific demands of certain applications. For instance, wearable devices require flexible materials with robust mechanical properties that can be seamlessly integrated with other fabrics, a requirement that many traditional materials do not satisfy. Consequently, there is an urgent need to develop high-performance materials for thermal applications.

Since their discovery in 2011 by Gogotsi et al., MXenes have received a lot of attention for their excellent performance. MXenes represent a new class of 2-dimensional (2D) transition metal carbides, nitrides, or carbonitrides [9]. The chemical formula of MXenes is M_*n*+1_X*_n_*T*_x_* (where *n* =1 to 4), where M represents early transition metals, X stands for carbon or nitrogen, and T refers to functional groups such as –F, –O, and –OH, which are incorporated into the material’s surface during the synthesis process.

MXenes are typically produced by selectively removing specific atomic layers from their precursor MAX phases through an etching process. The resulting multilayered MXenes can be further obtained using techniques such as ultrasonic oscillation or ball milling. Additionally, single-layer MXenes, which exhibit morphologies similar to graphene [10], can also be synthesized. However, unlike graphene’s single-atomic structure, MXenes consist of more stable M–X multilayers, such as M–X–M (3 layers, 211), M–X–M–X–M (5 layers, 312), and M–X–M–X–M (7 layers, 413), among others [11]. While the bonds between carbon atoms in graphene are purely covalent, the M–X bonds in MXenes exhibit a combination of covalent, ionic, and metallic characteristics. Furthermore, the wide variety of functional groups on the surface of MXenes can be tailored, indicating that M–X 2D materials (MXenes) possess more diverse and adjustable properties compared to graphene.

In 2014, Khazaei et al. [12] predicted the exceptional thermoelectric properties of functionalized Mo_2_C MXene. Since then, researchers have investigated various thermal properties of MXenes, including photothermal conversion, thermal conductivity, and thermal camouflage. These contributions have established the foundation for the application of MXenes in the thermal energy sector, creating new development opportunities and addressing challenges in material technology.

Although there is a growing body of research focused on the application of MXenes in the thermal energy sector, reviews dedicated to more specialized subfields, such as photothermal applications, remain relatively scarce and do not comprehensively cover the diverse applications of MXenes. This paper aims to provide an extensive overview of recent advancements in MXene preparation techniques and their functional applications across various thermal energy domains, including biomedical applications, solar desalination, wearable heaters, and thermoelectric systems. Finally, this review identifies the challenges and opportunities that MXenes and their composites encounter within the thermal energy landscape, offering new directions for future research and development in this field.

## Preparation of MXenes

MXenes are primarily produced via the selective etching of A atoms from their respective MAX phase materials [13,14]. During the etching process, the A layer is efficiently removed due to the comparatively weaker bonding strength of the M–A bonds in relation to that of the M–X bonds. This allows for the preservation of the M–X structure while producing the final MXene material [15,16]. Currently, several methods are employed to prepare MXenes, including hydrofluoric acid (HF) etching, lithium fluoride (LiF) and hydrochloric acid (HCl) etching, molten salt etching, strong acid/alkali etching, electrochemical etching, and chemical vapor deposition (CVD).

The properties of MXenes synthesized through various methods can differ greatly, making it essential to choose the most suitable preparation technique based on specific application requirements. Furthermore, the current synthesis methods produce MXenes with different terminal groups on their surfaces, such as –F and –OH, which importantly influence their performance characteristics [17]. Modifying these surface groups can yield MXenes with specific properties, thereby enhancing functionality for diverse applications [18]. This section provides an overview of the current methods for synthesizing MXenes and modifying their surface groups.

### Etching and exfoliation

In 2011, Naguib et al. [19] produced the first MXene by utilizing HF as an etchant to etch Ti_3_AlC_2_ into Ti_3_C_2_T*_x_* nanosheets. This method is highly effective for producing well-defined and uniformly spaced MXene layers, facilitating the synthesis of various MXene materials, such as Ti_2_CT*_x_* [20], Nb_2_CT*_x_* [21], V_2_CT*_x_* [22], and Nb_4_C_3_T*_x_* [23]. Moreover, it is essential to recognize that HF etching is especially effective for the preparation of MXenes when the A layer of the MAX phase consists of Al and Si, due to its high efficiency. However, HF is a highly corrosive and toxic acid, which presents considerable hazards to both human health and the environment during large-scale applications. Furthermore, MXene layers produced via HF etching may exhibit defects, such as pores [24], which can hinder subsequent modifications and applications. Therefore, careful control of HF concentration, etching temperature, reaction duration, and precursor particle size is essential for the preparation of high-quality MXenes.

An alternative approach involves the use of fluorine-containing acid solutions as etchants, providing a milder method for preparing MXenes. In 2014, Gogotsi and Barsoum pioneered the application of a mixed solution of LiF and HCl to etch Ti_3_AlC_2_, successfully producing Ti_3_C_2_T*_x_* with a large lateral size and minimal defects [25]. Moreover, other combinations of fluoride sources, such as potassium fluoride (KF), sodium fluoride (NaF), iron trifluoride (FeF_3_), and ammonium fluoride (NH_4_F) with HCl, have also been investigated. The duration of etching and the concentration of the etchant play a crucial role in determining both the yield and quality of the resultant MXenes. During the etching process, cations such as Li^+^ can infiltrate the interlayers of the MXenes, resulting in an expansion of interlayer spacing and a reduction in interlayer interactions. Consequently, high-quality, high-yield single-layer or few-layer MXene nanosheets with large lateral dimensions can be produced simply through hand-shaking or ultrasonic exfoliation. This approach circumvents the direct use of HF, thereby mitigating safety risks associated with the preparation process. Due to its favorable safety profile, this method has emerged as the predominant method for MXene synthesis.

However, it is important to recognize that MXene products prepared via this method will inevitably contain –F terminal groups, which may complicate surface modification and could present potential hazards to humans and the environment.

Molten fluoride salt etching is a well-established technique for producing MXenes. In 2016, Urbankowski et al. [26] reported the preparation of Ti_4_N_3_T*_x_*, the first 2D transition metal nitride, by selectively etching Al from Ti_4_AlN_3_ precursor powder using molten fluorine salt (KF + NaF + LiF). This innovative approach facilitated the subsequent acquisition of single-layer and few-layer Ti_4_N_3_T*_x_* through intercalation with tetrabutylammonium hydroxide, followed by ultrasonic exfoliation—a important breakthrough in the advancement of MXene materials. However, this method does introduce fluoride impurities during the preparation process, which necessitate removal using sulfuric acid later on, thereby increasing the complexity of the procedure. Moreover, the stripped Ti_4_N_3_T*_x_* nanosheets may contain titanium dioxide, which could potentially impact the future applications of the MXene material.

**Fig. 1.**
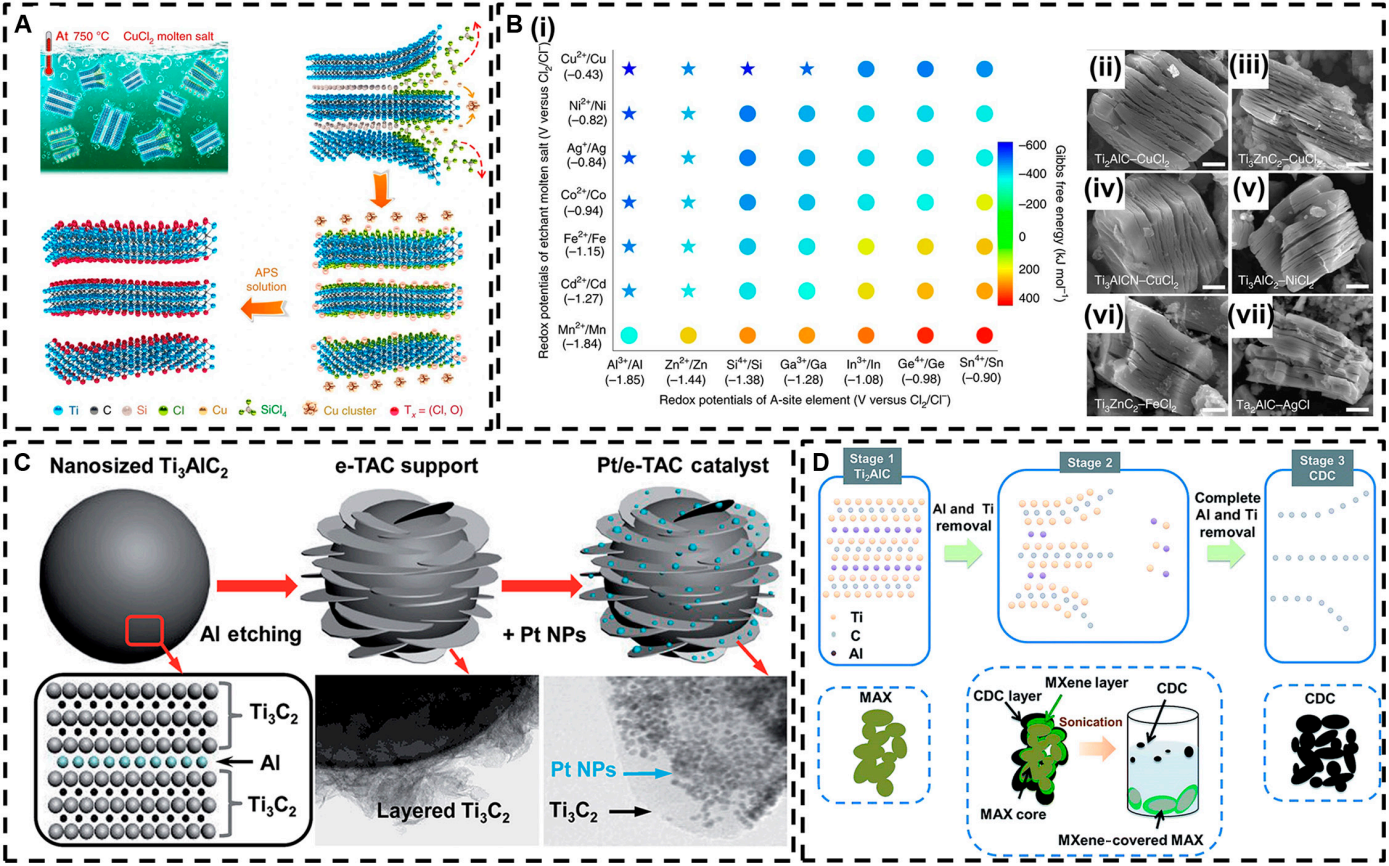
(A) Ti_3_C_2_T*_x_* MXene was prepared by the molten salt method using the Ti_3_SiC_2_ MAX phase as a precursor. (B) (i) Gibbs free energy mapping (700 °C); (ii to vii) MXenes from different MAX phases etched with Lewis acid chlorides typically exhibit an accordion-like morphology. Reproduced with permission from [27]. Copyright 2020, Springer Nature. (C) Schematic illustration of the preparation process for the Pt/e-TAC catalyst. Reproduced with permission from [29]. Copyright 2014, Royal Society of Chemistry. (D) Mechanism for electrochemical etching of Ti_2_AlC. Reproduced with permission from [31]. Copyright 2017, Royal Society of Chemistry. NPs, nanoparticles; APS, ammonium persulfate; CDC, carbon-dominated compound; e-TAC, surface Al etched Ti_3_AlC_2_.

The methods previously discussed inevitably introduce –F terminal groups during the synthesis process, which can negatively impact the adsorption capabilities of MXenes and present potential environmental and health hazards. Consequently, researchers have developed fluorine-free etching methods. For instance, in 2019, Huang’s group [27,28] discovered that the Ti_3_AlC_2_ undergoes a important reaction in molten zinc chloride (ZnCl_2_) salt, where Zn^2+^ cations act as Lewis acids, effectively replacing the A-site elements in MAX phases and resulting in the formation of a new MAX phase, Ti_3_ZnC_2_. Meanwhile, Cl^−^ anions perform a function similar to that of F^−^ by coordinating with M atoms. This etching method has been successfully extended to various Lewis acid chlorides, such as ZnCl_2_, FeCl_2_, and CuCl_2_, as well as to a broader array of MAX phase members that include A elements like Al, Zn, and Ga. By constructing a Gibbs free energy mapping diagram to evaluate the cation and A element redox potential/displacement reactions in a high-temperature molten salt environment, a universal strategy for synthesizing MXenes via Lewis acid molten salt etching of MAX phases has been proposed (Fig. 1A). This approach allows for the determination of the electron-donating ability of A-site elements in relation to Lewis acid salts, facilitating the selection of suitable Lewis acid molten salt and MAX phase systems for MXene preparation (Fig. 1B (i)). Experimental results indicate that various MAX phases can be effectively delaminated into their corresponding MXenes through suitable treatment with chloride molten salts (Fig. 1B (ii)). Consequently, the Lewis acid molten salt delamination method successfully yields fluorine-free MXenes, greatly improving the chemical safety of the experimental process while reducing the complexity and costs associated with waste liquid disposal.

Furthermore, some researchers have employed highly alkaline or acidic solutions to etch MAX phases, thereby generating MXene products. For instance, Xie et al. [29] immersed Ti_3_AlC_2_ in a sodium hydroxide (NaOH) aqueous solution at 80 °C for 100 h, followed by immersion in sulfuric acid (H_2_SO_4_) at the same temperature for 2 h. This process resulted in the formation of MXenes with sizes on the order of a few nanometers, which can serve as advanced carriers for platinum (Pt) catalysts (see Fig. 1C). While this method has greatly improved the resulting material’s corrosion resistance and conductivity, it primarily etches away only the surface Al layer to a depth of a few nanometers, leaving the internal MAX phase largely intact and resulting in poor etching effects.

To overcome the challenges posed by traditional etching methods, a novel electrochemical etching process has been developed, which is driven by charge transfer from the target material (A atom) to other species [30]. In 2017, Sun et al. [31] first demonstrated that MXenes, specifically Ti_2_CT*_x_*, could be obtained from Ti_2_AlC via electrochemical etching in a HCl electrolyte (Fig. 1D). Unlike traditional chemical etching methods that use HF or LiF/HCl, this approach yielded MXenes characterized by –Cl, –OH, and –O terminal groups without the introduction of fluorine ions. The findings indicated the formation of a 3-layer structure within the porous Ti_2_AlC during the electrochemical etching process, consisting of an outer layer of carbon-dominated compound, the MXene layer, and the unetched MAX phase. Further isolation of MXenes can be achieved through ultrasonic treatment in a water bath. These findings effectively illustrate that electrochemical etching can produce MXenes devoid of –F terminal groups. Moreover, Peng et al. [32] introduced an enhanced thermally assisted electrochemical etching technique that is applicable for the synthesis of various MXenes, including Ti_2_CT*_x_*, Cr_2_CT*_x_*, and V_2_CT*_x_*. The incorporation of appropriate heating was found to enhance etching efficiency, allowing for the avoidance of harmful etchants and minimizing associated issues such as surface damage and the presence of various terminal groups on the final product.

CVD is another widely used method for the synthesis of MXenes under nonfluoride conditions, involving gas-phase reactions at elevated temperatures. In the CVD process, a substrate is immersed in a metal salt solution, leading to the formation of a textured structure that arises from the electronic disparities among the metal atoms present on the surface [33]. In 2015, Yin et al. [34] developed a CVD method that successfully produced 2D ultrathin α-Mo_2_C crystals, which exhibited superconducting transitions and strong anisotropy in magnetic fields. By adjusting the CVD conditions, various 2D structures of α-Mo_2_C crystals can be synthesized. For example, high temperatures favor the growth of thicker MXene layers, while lower temperatures promote lateral growth, facilitating the production of large-area MXene nanosheets [35]. Consequently, CVD thus represents a versatile preparation method that offers the necessary conditions for the fabrication of various high-quality 2D MXene materials.

### Surface modification

The terminal groups on the surfaces of MXenes greatly influence their properties. Therefore, the rational design of surface functional groups and the adjustment of surface chemistry are crucial for expanding the applications of MXenes.

Targeted modification and optimization of MXenes through surface chemistry methods can mitigate their limitations and maximize their performance. For example, MXenes are highly susceptible to oxidation and exhibit considerable instability under environmental conditions, which severely restricts their practical applications. Li et al. [36] observed variations in the content of various elements and by-products during the oxidation process of Ti_3_C_2_T*_x_*, which was prepared using LiF and HCl. They found that environmental instability arises from numerous active oxidation sites on the surface, which were closely linked to the accumulation of etching by-products. To mitigate the formation of these by-products, researchers developed a slow etching method that employs tetramethylammonium hydroxide as the sole etchant, thereby avoiding the adsorption of fluorine-containing by-products. Finally, the Ti_3_C_2_T*_x_* flakes exhibited exceptional environmental stability, maintaining their original condition even after 4 months of dispersion in water. This enhanced stability facilitates their application in solar-powered desalination of seawater, demonstrating remarkable desalination capabilities with a maximum sodium ion rejection rate of 62.2% and a maximum water flux increase of 300%.

Additionally, traditional MXenes possess numerous hydrophilic functional groups on their surfaces, which can hinder their application in hydrophobic environments. Yang et al. [37] highlighted the metallic conductivity, high carrier mobility, and excellent capacitance properties of MXenes, suggesting that these materials are promising electrocatalysts for electrochemical nitrogen reduction reactions. Nevertheless, the hydrophilic characteristics of MXenes and the high proton affinity of their terminal groups often result in the hydrogen evolution reaction becoming the dominant reaction, masking the catalytic active sites for nitrogen binding [38]. To address this issue, researchers functionalized the surface of MXenes—specifically the abundant –OH groups—using condensation chemistry to create a thin hydrophobic molecular layer that regulates the local microenvironment. The results demonstrated that this hydrophobic layer, when used as an electrocatalyst, significantly restricted proton transfer during the electrochemical nitrogen reduction reaction process, promoted the exposure of active sites, and enhanced the chemical adsorption capacity of N_2_, leading to a substantial increase in the NH_3_ production rate. Under the same conditions, the faradaic efficiency and NH_3_ yield of the induced current were found to be 3.5 and 6.5 times higher, respectively, than those of the original Ti_3_C_2_T*_x_*.

In summary, various preparation methods yield MXene materials with distinct surface structures, and these differences importantly influence their chemical properties. The surface structure of MXenes plays an important role in determining their electronic characteristics, stability, and catalytic performance; therefore, selecting the appropriate preparation method is essential. By optimizing the preparation process according to specific application requirements, the surface structure and chemical properties of MXenes can be tailored to produce materials with exceptional characteristics. For instance, in the domains of energy storage, catalysis, and sensing, different preparation methods can affect the specific surface area, electrical conductivity, and chemical reactivity of MXenes. Consequently, the development of diverse preparation techniques and the careful selection of the most suitable method based on specific needs are vital for realizing the practical application potential of MXenes.

## MXene Materials for Thermal Applications

Since the emergence of MXene materials in 2011, researchers have continuously explored their potential across various fields of application. Currently, MXenes exhibit great promise in electricity, thermodynamics, magnetism, and more. Among these areas, there has been a relatively greater emphasis on the study of MXenes in the domains of electricity and magnetism. However, as nonrenewable energy resources like natural gas and oil become depleted, the severity of the energy crisis has intensified, making the development of alternative energy sources increasingly urgent. Solar energy, being abundant and renewable, offers a promising solution to help alleviate the energy crisis. This section focuses on the advancements in research involving MXenes and their composite materials in the fields of photothermal, photoelectric, and thermal conduction applications.

### Thermal energy conversion

#### Photothermal conversion

Photothermal conversion is defined as the process of harnessing solar radiation energy through methods such as reflection and absorption, subsequently converting it into heat for various applications [39–42]. An ideal photothermal material is characteristic by its ability to efficiently absorb a broad spectrum of solar radiation while also exhibiting high photothermal conversion efficiency. Researchers have developed various nanostructured photothermal materials, including semiconductors [43], nanocarbon materials [44], MXenes [45], and specific organic polymers [46]. Under solar radiation, these materials absorb photons from sunlight, undergo photoexcitation, and facilitate electron migration, ultimately converting light energy into thermal energy.

**Fig. 2.**
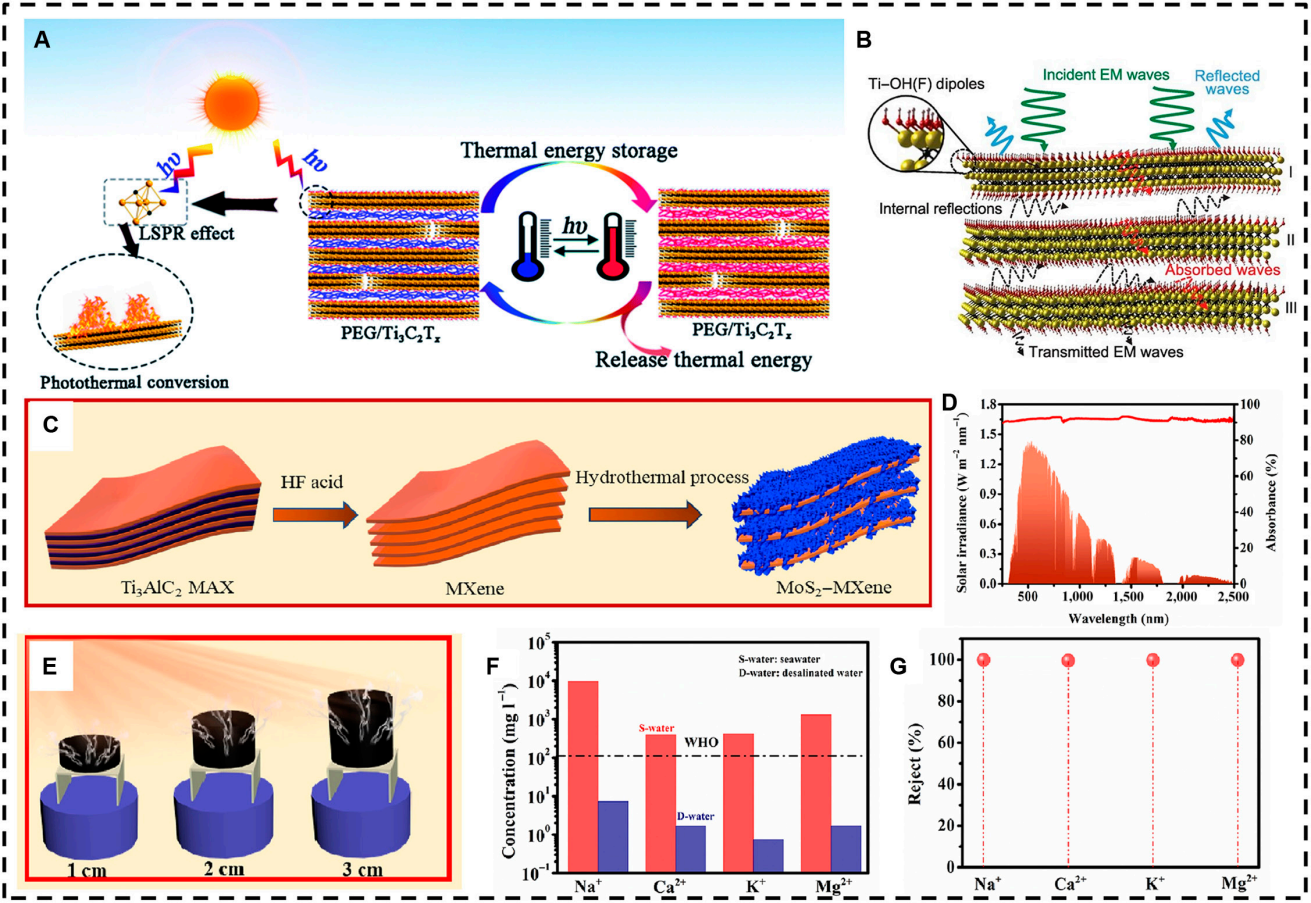
(A) The localized surface plasmon resonance (LSPR) effect exhibited by MXenes contributes to the exceptional photothermal conversion and thermal storage capabilities of polyethylene glycol (PEG)/Ti_3_C_2_T*_x_* composites. Reproduced with permission from [117]. Copyright 2019, Royal Society of Chemistry. (B) The electromagnetic interference (EMI) shielding mechanism of MXenes, with a focus on Ti_3_C_2_T*_x_* as a representative example. Reproduced with permission from [49]. Copyright 2016, The American Association for the Advancement of Science. (C) The synthetic process of MoS_2_–MXene. (D) Light absorption characteristics of the MoS_2_–MXene film across the entire solar spectrum. (E) Schematic diagram of the photothermal system featuring 3-dimensional (3D) evaporators. (F and G) Desalination performance of the 3D-PF system. Reproduced with permission from [57]. Copyright 2022, Elsevier Ltd. EM, electromagnetic; WHO, World Health Organization; PF, paraffin.

The unique structure of MXene materials endows them with a strong localized surface plasmon resonance effect within the solar spectrum (Fig. 2A), a characteristic typically observed in metal nanoparticles [47,48]. This distinctive property renders MXenes highly absorbent and efficient at converting solar energy into heat, a capability that other 2D nanomaterials, such as graphene, lack. While the specific mechanisms underlying MXenes’ photothermal conversion are still under exploration, their outstanding performance has already attracted great attention from researchers.

Shahzad et al. [49] noted that MXenes exhibit an electromagnetic interference (EMI) shielding capability, which facilitates the transmission of electromagnetic waves through the MXene lattice structure. These waves subsequently experience internal reflection among the layers and are ultimately absorbed by the material (see Fig. 2B). This property ensures effective light absorption across a wide range of solar spectra. In their experiments, Li et al. [50] employed a droplet light absorption and thermal measurement system to accurately assess the photothermal conversion efficiency of MXenes. Their results indicated that the conversion efficiency of MXenes approached 100% across all wavelengths of the light source, thereby fully demonstrating the exceptional photothermal conversion capabilities of these materials.

This section specifically focuses on the applications of MXenes in solar seawater desalination and biomedicine, emphasizing their potential to advance photothermal conversion technologies.

##### Solar water desalination

Utilizing solar energy for seawater desalination represents an effective approach to resource conservation. Interface evaporation solar energy desalination is an efficient, eco-friendly, and practical method for addressing freshwater shortages. High-quality light-absorbing materials are essential, as they not only minimize heat loss during seawater evaporation but also prevent salt accumulation, thereby protecting equipment and enabling operation under various harsh conditions. MXenes have attracted considerable interest owing to their remarkable capabilities in photothermal conversion [51–54]. However, the relatively smooth surface of MXenes results in high surface reflectance, which can impede their direct application in solar seawater desalination. Consequently, developing composite materials that incorporate additional substances to modify the surface microstructure offers a promising strategy for enhancing the light absorption of MXenes and improving the efficiency of maximizing the photothermal conversion [55,56].

For instance, Guo et al. [57] synthesized a flexible MoS_2_–MXene thin film using a one-pot hydrothermal method, as illustrated in Fig. 2C. This film exhibited over 90% light absorption across the entire solar spectrum (Fig. 2D), indicating its capability for broad-spectrum light absorption. The optical–thermal system depicted in Fig. 2E measured the evaporation rate of the thin film, which reached an impressive 2.5 kg·m^−2^·h^−1^. Importantly, when the surface temperature of the evaporator stabilized, the water temperature remained constant, confirming the thermal stability of the thin film. Furthermore, the research team assembled a 3-dimensional (3D) evaporator using a MoS_2_–MXene@PF (PF: paraffin) layer. This configuration resulted in a favorable gradient heating effect and provided a crystallization field for salt during the seawater evaporation process, leading to nearly complete elimination of salt crystals on the evaporator surface. The evaporation rate remained consistent at 3.12 kg·m^−2^·h^−1^ even after multiple cycles. Subsequent tests revealed that the resulting ion concentration in the water was reduced to 99.5%, demonstrating excellent desalination performance (Fig. 2F and G).

Future research should concentrate on integrating photothermal materials into intelligent systems that can adaptively respond to environmental changes. For instance, materials capable of adjusting their photothermal efficiency in response to varying light intensities or temperatures will enhance the functionality of both energy conversion and thermal management systems. Such innovations could lead to advancements in smart textiles, responsive coatings, and dynamic thermal regulation devices. The transition from laboratory-scale production to scalable manufacturing remains a critical challenge. Advances in fabrication techniques, such as 3D printing, roll-to-roll processing, and spray coating, can facilitate the mass production of photothermal materials. These methods not only improve scalability but also lower costs, making photothermal technologies more accessible for commercial applications.

##### Biomedical applications

MXenes were initially developed for photothermal applications in the biomedical field, and they have garnered attention for several key characteristics [58]. First, unlike inorganic quantum dots (QDs) and hydrophobic nanoparticles, MXenes exhibit excellent hydrophilicity, making them suitable for biomedicine. Second, MXenes contain essential elements for living organisms, including C and N, while the early transition metals present in MXenes are relatively inert to biological systems, rendering them relatively nontoxic and biocompatible [59]. Third, MXenes possess excellent absorption characteristics in the near-infrared (NIR) region [60], which facilitates their application in in vivo photoacoustic imaging and photothermal therapy (PTT) within the first or second biological windows [61]. Finally, the abundant functional groups on the surface of MXene materials can be modified through various surface-functionalization techniques, establishing a foundation for diverse applications in advanced biomedical fields [62]. Currently, the application of MXenes in biomedicine focuses mainly on 4 areas: antibacterial materials, biomedical imaging, tumor therapy, and biosensors. In addition to biomedical imaging, the other 3 areas are directly related to the photothermal conversion effect of MXenes.

The emergence of various 2D nanomaterials has generated optimism for the development of highly efficient antibacterial agents [63]. The antibacterial mechanisms of these innovative 2D nanomaterials primarily include increasing transmembrane permeability, inducing cell membrane rupture, reducing metabolic activity, damaging DNA, and inflicting physical harm to the cell membrane through the sharp edges of the materials [64]. Owing to their comparatively high specific surface area, ease of surface modification, and ability to incorporate diverse antibacterial functional groups, MXenes are emerging as a stable and long-lasting antibacterial agent [65].

**Fig. 3.**
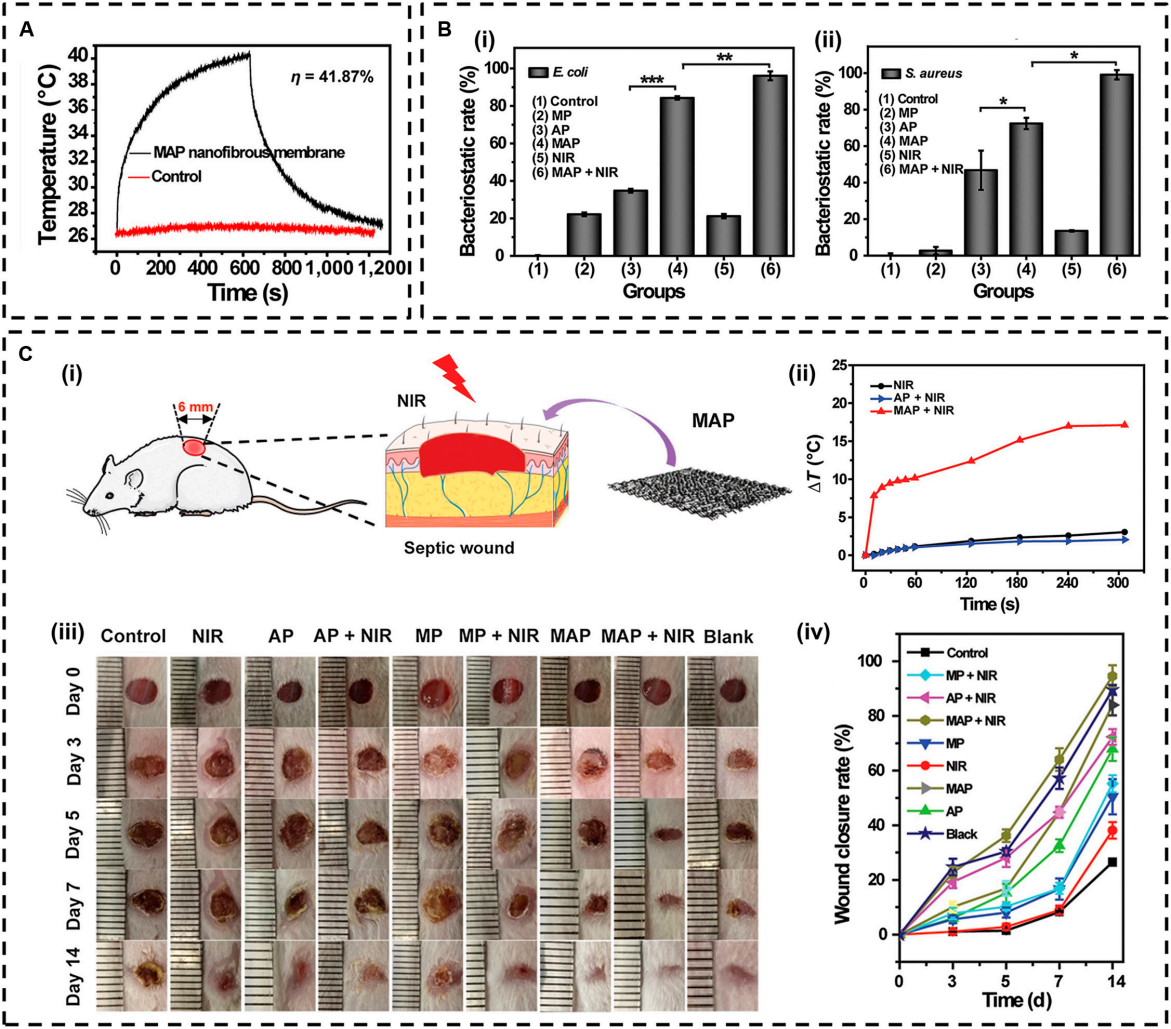
(A) Steady-state heating curves of the MXene–amoxicillin–polyvinyl alcohol (MAP) nanofibrous membrane and saline. (B) Antibacterial efficacy of the nanofibrous membranes against (i) *Escherichia coli* and (ii) *Staphylococcus aureus*, where * means significant difference: * indicates *P* < 0.05, ** means *P* < 0.01, and *** means *P* < 0.001. (C) In vivo antimicrobial testing: (i) experimental flow diagram; (ii) temperature increase in the *E. coli* wound; (iii) wound pictures and (iv) wound healing rates within 14 d under various conditions. Reproduced with permission from [66]. Copyright 2021, Elsevier Ltd. NIR, near-infrared; MP, MXene–polyvinyl alcohol; AP, amoxicillin–polyvinyl alcohol.

Electrospun nanofiber membranes, characterized by their 3D network structure, can maintain moisture balance at wound sites and promote healing, making them promising candidates for wound dressings. Xu et al. [66] synthesized amoxicillin (AMX), MXene, and polyvinyl alcohol (PVA) into an antibacterial MXene–AMX–PVA (MAP) nanofiber film using electrospinning techniques. MXene has the ability to convert NIR lasers into heat, thereby generating a localized high-temperature environment that facilitates the release of AMX [67]. Due to the presence of MXene, the MAP nanofiber film exhibited a high photothermal conversion efficiency of 41.9% (Fig. 3A), surpassing that of traditional photothermal agents (PTAs) such as gold nanorods and bismuth nanoparticles. In vitro antibacterial experiments demonstrated that MAP possessed significant antibacterial properties, with inhibition rates for *Escherichia coli* and *Staphylococcus aureus* increasing to 99.61% and 99.1%, respectively, when treated with MAP in combination with NIR laser (Fig. 3B). These findings suggest that the photothermal conversion capabilities of MXene can effectively improve the antibacterial activity of the MXene membrane. Furthermore, in vivo antibacterial experiments revealed that the MAP + NIR group exhibited the highest temperature increase, and the wounds of mice treated with this combination showed the best healing rates (Fig. 3C). This highlights the potential of MAP nanofiber membranes as effective wound dressings. Biosafety analyses further indicated the MAP nanofiber membrane’s great potential for use as an antibacterial dressing for wound care, underscoring its importance in the applications of electrospun nanofiber membranes for wound healing and infection management.

The exceptional antibacterial and antifouling properties of MXenes, combined with their effective film-forming capabilities [25], have motivated researchers to develop MXene-based antibacterial membranes for wastewater treatment [68–70]. The future holds promise for the development of effective MXene antibacterial materials.

In recent years, the application of MXenes in tumor treatment has become a prominent focus of biomedical research. Since 2017, numerous MXene-based composite materials have been developed to effectively target tumors, which continue to pose a great threat to human health. Despite ongoing efforts, there remains a critical need for effective, safe, and feasible treatment options.

PTT is a noninvasive treatment that employs photothermal materials to convert light energy into localized hyperthermia, effectively destroying cancer cells. PTAs are a crucial component of this therapy, as they transform light into heat to ablate tumors. Research has demonstrated that PTAs with strong NIR-II absorption are particularly effective for treating deep-seated tumors. MXene materials, such as Ti_3_C_2_T*_x_* [71], Nb_2_CT*_x_* [72], Ta_2_C_3_T*_x_* [73], and Ti_3_CNT*_x_* [74], are promising PTAs that exhibit strong light absorption properties and high photothermal conversion efficiency across a broad spectral range, from ultraviolet to NIR. This versatility makes them ideal candidates for cancer treatment.

**Fig. 4.**
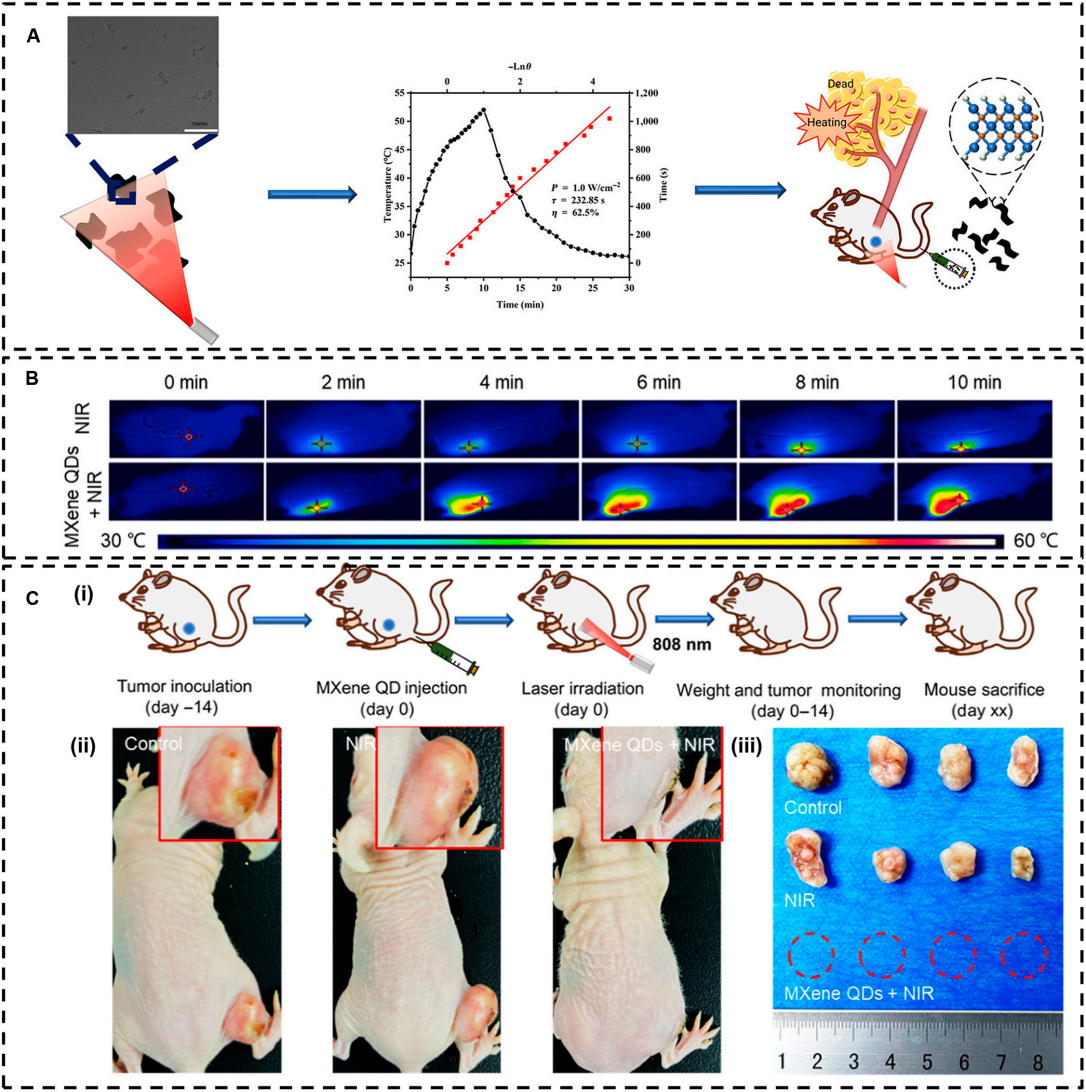
(A) Ti_3_C_2_T*_x_* MXene quantum dots (QDs) exhibited high photothermal conversion efficiency and were utilized as photothermal agents (PTAs) in cancer therapy. (B) Following a 4-h injection period, infrared photothermal imaging of the tumor site was performed within 10 min using an-808 nm laser (1 W/cm^2^) for irradiation. (C) Photothermal therapy (PTT) experiment of MXene QDs in mice: (i) experimental flow diagram, (ii) typical photographs of mice, and (iii) tumors under various treatment conditions after 14 d. Reproduced with permission from [75]. Copyright 2024, American Chemical Society.

Hu et al. [75] synthesized Ti_3_C_2_T*_x_* MXene QDs, which exhibited a photothermal conversion efficiency of up to 62.5% under 808-nm laser irradiation, making them suitable as PTAs for tumor therapy (Fig. 4A). Experimental results demonstrated that, compared to that with pure NIR irradiation, the temperature at the tumor site in mice injected with MXene QDs was significantly higher under the same irradiation duration, resulting in effective tissue tumor ablation (Fig. 4B). The findings from the PTT experiments indicated that mice in the MXene QDs + NIR laser irradiation group experienced only mild edema in the early stages of the experiment, which resolved within 2 d. In contrast, the tumors in the control group and in mice exposed solely to NIR laser irradiation continued to grow in size (Fig. 4C). Furthermore, the body weight of mice in the MXene QD group did not exhibit significant changes, suggesting that MXene QDs possess excellent biosafety and can effectively serve as PTAs for PTT. For other classes of MXenes, bovine serum albumin-modified d-Ti_3_CNT*_x_* demonstrated remarkable tumor inhibition at the NIR-I and NIR-II biological windows, enabling tumor-free treatment at 1,064 nm with minimal side effects [74]. These studies underscore the importance of rational design of MXene-based PTAs at the atomic level. Given this innovative approach and its potential applications, it is anticipated that the development of MXene materials for tumor therapy will continue to expand and evolve.

MXenes exhibit exceptional photothermal conversion capabilities and are widely utilized in biosensors [76]. In particular, their remarkable flexibility presents important potential for applications in wearable biosensors. Chao et al. [77] combined Ti_3_C_2_T*_x_* nanosheets with silver nanowires to leverage their efficient NIR absorption and photothermal conversion properties, resulting in the development of a wearable flexible electronic sensor. This sensor could achieve the highest saturation temperature within 90 s under the irradiation of an external light source, and it returned to room temperature within 60 s after the light source was turned off. Upon exposure to an external light source, the temperature rapidly reverted to the highest saturation temperature, demonstrating excellent sensitivity and cycle stability. The saturation temperature of this sensor was closely linked to the power of the external NIR light source, allowing for effective temperature control by adjusting the light source’s power. Furthermore, this sensor exhibited outstanding sensing performance, biocompatibility, and antibacterial properties, making it a promising candidate for wearable devices in hyperthermia, human health monitoring, and other applications. Notably, the additional advantageous properties of MXenes also suggest great potential for applications in chemical sensors, including gas sensors [78] and pressure sensors [79].

The potential of photothermal conversion in biomedical applications extends far beyond traditional therapeutic methods, offering innovative solutions for targeted treatment and diagnosis. One promising direction is the development of multifunctional nanomaterials that can act as both PTAs and imaging contrast agents, enabling real-time monitoring of treatment effectiveness. Additionally, integrating PTT with immunotherapy strategies could enhance the body’s immune response against tumors, facilitating a dual-action approach that not only targets cancer cells but also primes the immune system for long-term protection. Advances in biocompatible and biodegradable photothermal materials will pave the way for safer and more effective interventions, minimizing side effects and promoting the sustainable disposal of medical devices posttreatment. Furthermore, exploring photothermal conversion for applications such as wound healing, drug release, and hyperthermia treatment presents exciting opportunities, where localized heating can stimulate cellular responses or release therapeutic agents in a controlled manner. As research progresses, combining photothermal conversion with other therapeutic modalities may lead to synergistic effects that can help improve patient outcomes in oncology, regenerative medicine, and beyond.

#### Electrothermal conversion

Joule heating refers to the process of generating heat in materials through the application of low voltage to electronic devices, which is essential for maintaining a constant temperature necessary for device operation [80,81]. Joule heating devices, which convert electrical energy into thermal energy, have found widespread application in both personal and industrial settings, particularly in wearable technology. Wearable heaters demonstrate excellent potential for use in insulation, thermal therapy [82], defrosting, and sensing [83]. Considerable attention has been directed toward developing lightweight, comfortable, and breathable fabric-based wearable heaters to meet the growing demand for enhanced wearability [84,85]. Despite substantial advancements in fabric-based wearable heaters, challenges persist, including dependence on a single energy source, slow thermal response times, and limited heating temperature ranges. Furthermore, users of wearable heaters may face potential risks such as exposure to electromagnetic radiation, bacterial cross-contamination, and fire hazards resulting from thermal failures.

MXene materials, known for their metallic conductivity of up to 10^6^ S/m, can be seamlessly integrated into textiles, presenting great potential as Joule heating materials for fabrics, fibers, and films [86–88]. This positions them as a foundation for applications in wearable electronic devices. For example, Liu et al. [84] successfully developed a flame-retardant, antibacterial, and electromagnetic shielding dual electric/optical-driven polymer/MXene composite fabric heater. In this composite, MXene nanosheets were tightly bonded to the surface of polymer fibers through hydrogen bonding and physical insertion, preserving the fabric’s original breathability and comfort. With a MXene content of 17.3 wt%, the electrical conductivity of the M-textile fabric reached 117 S/m, enabling a wide heating range of 40 to 174 °C under low driving voltages of just 13.5 V. Remarkably, the M-textile fabric exhibited excellent durability, robustness, and heating stability, maintaining effective electrical heating performance after extensive cycling, severe deformation, and washing. Moreover, the M-textile fabric demonstrated a rapid and sensitive photothermal conversion response across various wavelengths (NIR, far-infrared, and natural light), achieving a heating temperature range of 40 to 204 °C while retaining good photothermal conversion characteristics after washing, deformation, and prolonged heating. In addition to these properties, the M-textile-based wearable heater showcased exceptional electromagnetic shielding capabilities, along with flame retardant and antibacterial qualities. These studies offer innovative solutions for rapid, safe, and efficient heat transfer in complex environments, underscoring the substantial potential of the M-textile heater in applications related to insulation, thermal therapy, deicing, fire prevention, and antibacterial fields.

Zhao et al. [89] further advanced this field by depositing Ti_3_C_2_T*_x_* nanosheets onto cellulose nonwoven fibers to create MXene-based multifunctional smart fabrics. The metallic-like conductivity of the MXene enabled effective Joule heating, which could also facilitate the moderate elimination of bacteria surrounding wounds during infection healing. This multifunctional, smart, and flexible fabric is poised to play a vital role in the next generation of wearable electronic devices.

**Fig. 5.**
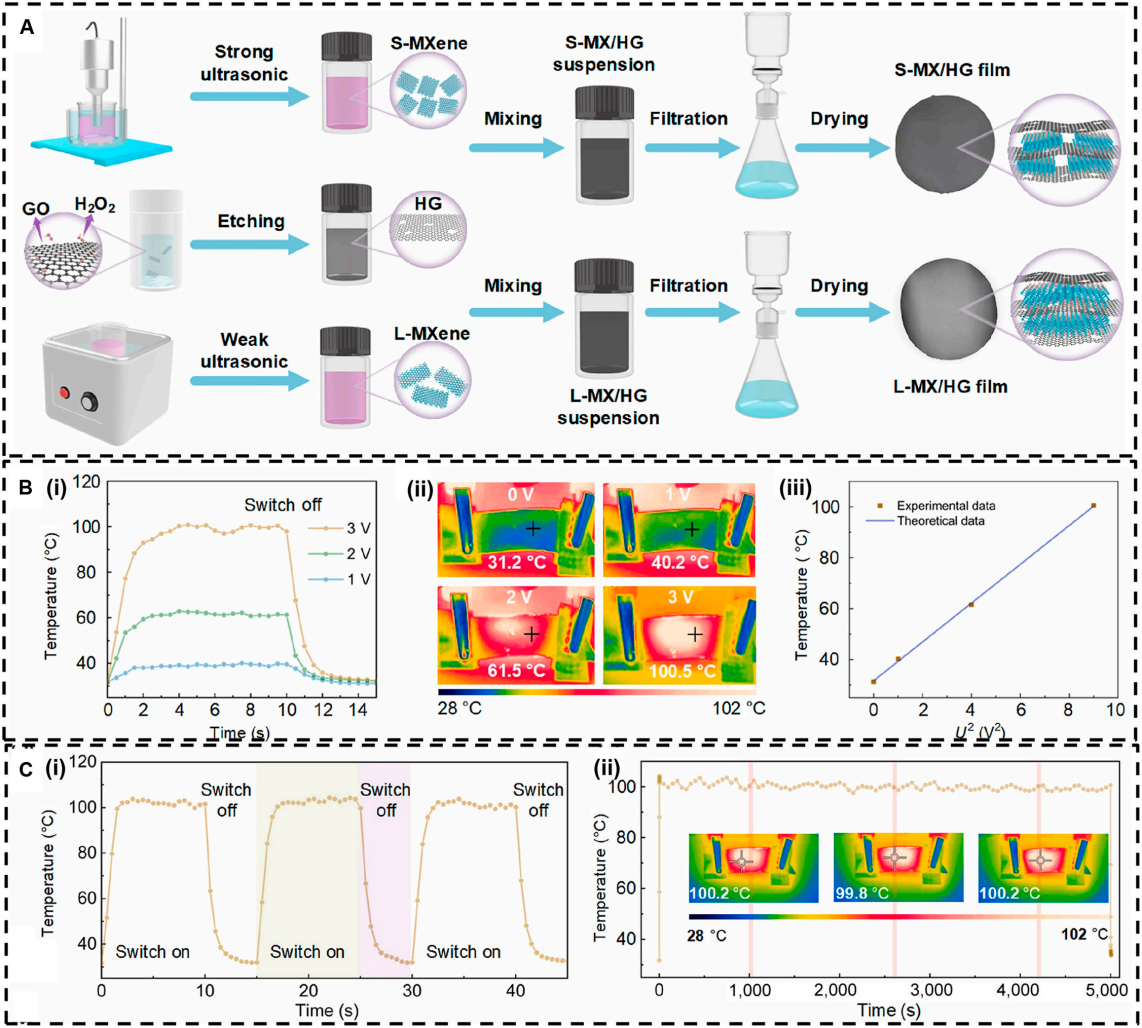
(A) Schematic representation of the fabrication process of MXene/holey graphene (MX/HG) films. (B) (i) Time-dependent surface temperature of the L-MX/HG film at various voltages; (ii) infrared images under different driving voltages; (iii) *T*–*U*^2^ plot of the L-MX/HG film. (C) (i) Cyclic test and (ii) stability test of the L-MX/HG film at a voltage of 3 V. Reproduced with permission from [90]. Copyright 2023, Elsevier Ltd. GO, graphene oxide; S-MXene, small-sized MXene; L-MXene, large-sized MXene; S-MX/HG, MX/HG with small-sized MXene; L-MX/HG, MX/HG with large-sized MXene.

MXene-based thin films represent a important category of materials for Joule heating applications. Cai et al. [90] prepared MXene/holey graphene (MX/HG) composite films utilizing a layered structure, as illustrated in Fig. 5A. The hydrogen bonding between the holey graphene and MXene sheets enhanced the mechanical properties of the composite and effectively prevented the self-aggregation of the MXene layers. In addition, the composite film exhibited excellent electrical conductivity. L-MX/HG with large-sized MXene and an HG content of 0.5 wt% achieved impressive conductivity levels of 9,800 S/m. This outstanding conductivity translated into exceptional low-voltage Joule heating performance. For instance, at 3 V, the surface temperature of the L-MX/HG film rose to 100 °C within just 4 s (Fig. 5B (i) and (ii)). Upon cutting off the voltage, the film was able to swiftly return to room temperature, indicating good sensitivity.

Furthermore, the L-MX/HG film demonstrated a strong linear relationship between temperature and the square of the voltage, affirming its adherence to the Joule heating effect (Fig. 5B (iii)). Moreover, the film exhibited excellent cyclic stability and thermal heating stability (Fig. 5C). The L-MX/HG film also showcased effective EMI shielding and photothermal conversion capabilities. These characteristics position the L-MX/HG film as a versatile candidate for a variety of applications.

Electrothermal conversion, which involves the direct transformation of electrical energy into thermal energy, presents exciting opportunities across various applications, particularly in energy efficiency, heating technologies, and sensing mechanisms. The advancement of materials with high electrical conductivity and thermal responsiveness, such as nanostructured materials and conductive polymers, could enable more efficient electrothermal systems that minimize energy loss during conversion. These materials could be integrated into smart heating systems for building applications, providing precise temperature control while enhancing energy efficiency. Moreover, electrothermal conversion technologies are being explored for innovative thermal management solutions in electronic devices, where controlled heating can prevent overheating and prolong device lifespan. In the realm of flexible electronics, the coupling of electrothermal conversion with responsive materials can create adaptive devices capable of adjusting their thermal output based on environmental conditions. Additionally, emerging applications in medical therapies, such as electrothermal ablation techniques, could facilitate minimally invasive procedures that precisely target diseased tissues with minimal collateral damage. As research continues to evolve, the integration of electrothermal systems with renewable energy sources like solar and wind could lead to breakthroughs in sustainable heating solutions, further enhancing the appeal of electrothermal conversion technologies in a rapidly changing energy landscape.

### Thermal conduction

In the 5G era, rapid advancements in science and technology have increased the demand for effective heat dissipation in various electronic components [91]. This situation necessitates the development of materials exhibiting high thermal conductivity to address the heat dissipation challenges associated with electronic devices. The layered structure of MXenes, characterized by atomic layer-by-layer stacking, strong intralayer atomic bonding, and weak van der Waals forces between layers, often results in anisotropic spatial configurations that exhibit excellent thermal conductivity. Furthermore, MXenes possess diverse surface functional groups that can be modified through various surface treatment techniques to enhance their physical and chemical properties [92,93].

Despite their excellent electrical and thermal properties, the actual performance of synthesized MXenes can fall short of expectations due to structural defects that arise during the synthesis process. Current research on the thermal conductivity of MXenes primarily focuses on addressing intrinsic defects within the materials and exploring their application as fillers in other matrix materials to enhance thermal performance [94,95]. For example, to mitigate this issue, Nguyen et al. [96] improved the titanium (Ti) defects in MXenes by utilizing platinum (Pt) gas-phase infiltration, which formed covalent Pt–C bonds that act as bridges between MXene sheets. This innovation resulted in enhanced thermal conductivity pathways, leading to increases of 1.8 times and 5 times in in-plane and cross-sectional thermal conductivity, respectively.

The unique layered structure of MXenes makes them advantageous for thermal conductivity applications, often rendering them suitable as fillers in polymer matrices. For example, Wang et al. [97] successfully prepared 3D MXene/polydimethylsiloxane composite materials with a 3D interconnected structure. They discovered that as the MXene skeleton became increasingly interconnected, the thermal conductivity of the composite improved rapidly with rising MXene content, particularly within the filler loading range of 0% to 3.5%. When the MXene content reached 2.5%, the thermal conductivity achieved a value of 0.576 W/(m·K), representing a 220% increase compared to that of pure polydimethylsiloxane. However, as the MXene content increased beyond this point, the thermal conductivity began to decline. Excess MXene sheets could obstruct alignment along the thickness direction, thereby reducing connectivity between the thin sheets in that dimension. Additionally, a high MXene loading complicated the vacuum-assisted impregnation process, potentially introducing gaps that obviously diminish thermal conductivity.

**Fig. 6.**
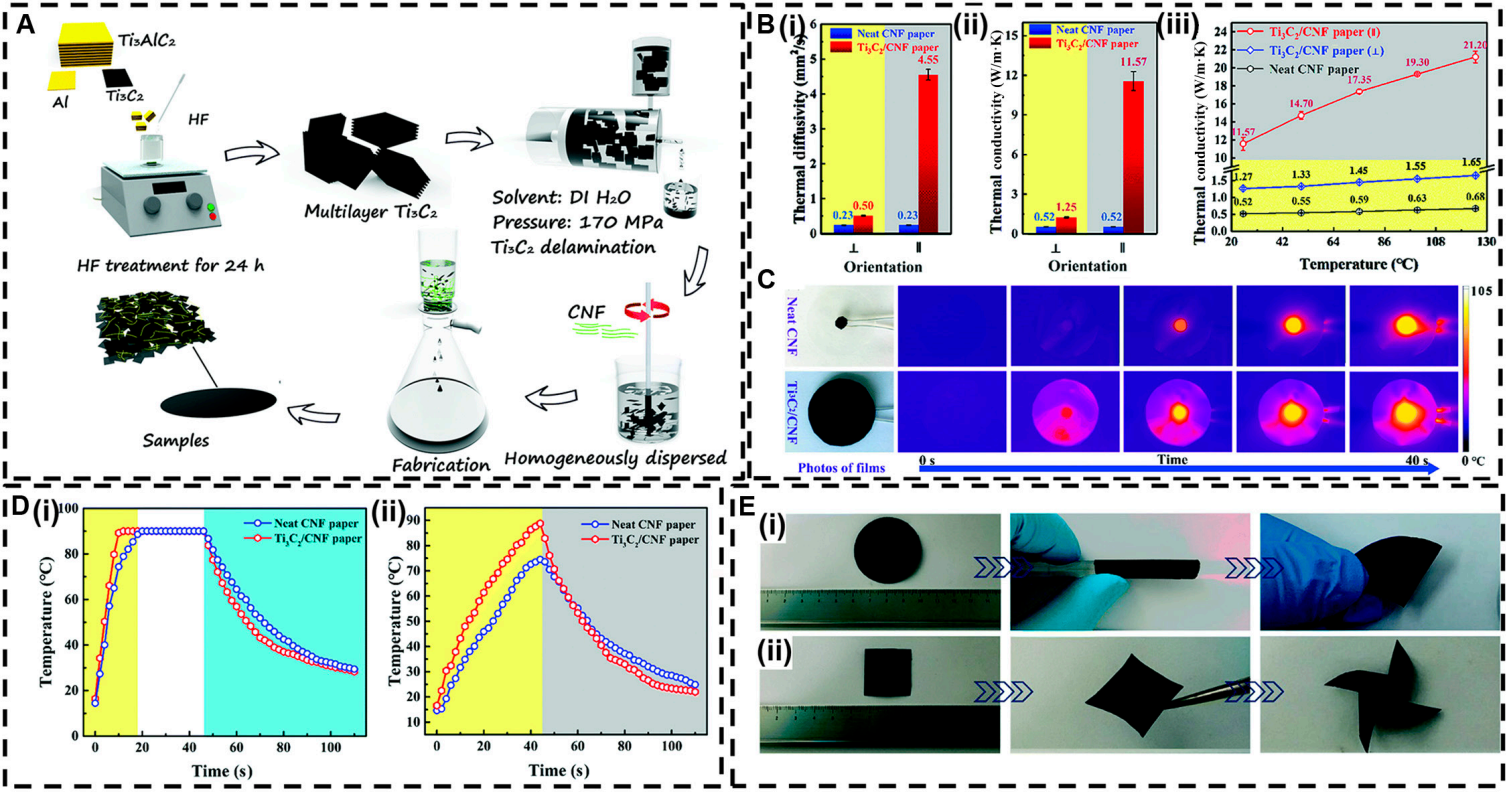
(A) Preparation process diagram of Ti_3_C_2_/cellulose nanofiber (CNF) composite films. (B) (i) Thermal diffusion and (ii) thermal conductivity of both the neat CNF film and Ti_3_C_2_/CNF composite film in various directions, as well as (iii) their changes with temperature. (C) Infrared images illustrating the neat CNF film and Ti_3_C_2_/CNF composite film after heating. (D) Temperature curves of the pure CNF film and Ti_3_C_2_/CNF composite film at (i) the center point and (ii) 5 mm from the center point. (E) The Ti_3_C_2_/CNF composite film provides excellent flexibility. Reproduced with permission from [98]. Copyright 2020, Royal Society of Chemistry. DI, deionized.

Compositing with other materials is an important way to improve the inherent defects of MXenes, which contributes to the wide application of MXenes in the field of thermal conductivity. Pure Ti_3_C_2_T*_x_* films, while exhibiting desirable properties, are relatively thin and prone to oxidation. By compositing Ti_3_C_2_T*_x_* with other materials, it is possible to improve both the mechanical strength and oxidation resistance of the films. Song et al. [98] investigated the water solubility of cellulose nanofiber (CNF) and its compatibility with Ti_3_C_2_ suspensions, both of which demonstrate good adhesion properties. They prepared several layers of Ti_3_C_2_ nanosheets through ultrahigh-pressure and low-temperature delamination, subsequently employing vacuum-assisted filtration to produce flexible CNF/Ti_3_C_2_ composite films (Fig. 6A). The diffusivity of the Ti_3_C_2_/CNF paper was anisotropic compared to that of pure CNF paper (Fig. 6B (i)). When the load was 50 wt%, the in-plane and out-of-plane thermal conductivities of the Ti_3_C_2_ heat sink/CNF paper were 140% and 2,130% higher, respectively, than those of pure CNF paper (Fig. 6B (ii)). This significant enhancement was attributed to the layered structure of the Ti_3_C_2_ nanosheets embedded within the CNF matrix. Furthermore, the Ti_3_C_2_/CNF paper exhibited greater temperature sensitivity and maintained higher thermal conductivity at any given temperature compared to both pure CNF paper and Ti_3_C_2_ films (Fig. 6B (iii)). Consequently, the Ti_3_C_2_/CNF film demonstrated superior performance compared to conventional heat exchangers at relevant operating temperatures. It is noteworthy that this composite film, with its excellent in-plane thermal conductivity, also exhibited effective heat dissipation performance (Fig. 6C and D). The temperature curve illustrated the high-efficiency heat dissipation capability of the Ti_3_C_2_/CNF heat exchanger. Additionally, the synthesized Ti_3_C_2_/CNF composite film displayed remarkable flexibility (Fig. 6E), suggesting its potential application in flexible electronic devices.

Moreover, Lee and Kim [99] prepared a composite film by simultaneously integrating MXene and aggregated boron nitride, which was coated with polysilazane, into PVA. In this composite, polysilazane established a thermal conduction pathway, while MXene served as structural support, effectively enhancing thermal conductivity. Their results indicated that when the total filler content reached 44%, the in-plane and through-plane thermal conductivities measured 1.51 and 4.28 W/(m·K), respectively. In another approach, Jia et al. [100] utilized microencapsulation technology to prepare ammonium polyphosphate coated with MXene (MAPP), which was then combined with polyurethane (PU) and graphite fiber (GF) to form PU/GF/MAPP films. The thermal conductivity of the PU/GF/MAPP films increased by 59.3% compared to that of PU/GF films, while the electrical conductivity improved by 57.7%, indicating broad application prospects in advanced microelectronic systems.

The study of thermal conduction offers important opportunities for advancing materials science and engineering, particularly in the development of ultrathin films and nanostructured materials designed for enhanced thermal management applications. As industries strive for improved energy efficiency, understanding the mechanisms of heat transfer at the nanoscale will become increasingly crucial. Future research should focus on designing novel thermal conductors that exhibit exceptionally low thermal resistance while maintaining mechanical integrity, thereby enabling their use in a wide range of applications, from advanced cooling systems in electronics to thermal insulation materials in building construction. Additionally, exploring hierarchical structures that combine different materials to create tailored thermal conduction pathways could yield innovative solutions for heat dissipation and thermal regulation. The integration of phase change materials (PCMs) with high-thermal-conductivity substrates also holds promise for dynamic thermal management systems that can actively adapt to changing thermal loads. Furthermore, the interplay between thermal conduction and other transport phenomena, such as electrical conduction and fluid dynamics, may provide new insights that drive the development of multifunctional materials capable of addressing the increasing complexity of thermal management challenges in emerging technologies, including flexible and wearable devices, automotive applications, and renewable energy systems.

### Phase change thermal storage

Phase change thermal storage systems provide an effective solution for mitigating energy depletion [101], particularly by utilizing solid–liquid PCMs that possess excellent practical application value. Thermal energy storage technology is essential for enhancing energy utilization efficiency and protecting the environment. It addresses the imbalance between heat supply and demand and holds considerable promise across various sectors, including solar energy utilization, peak shaving and valley filling in electricity grids, waste heat recovery, and energy conservation in industrial settings, commercial buildings, and air-conditioning systems. The ideal heat storage material should exhibit a high capacity for solar energy conversion and excellent thermal conductivity [102,103]. Notably, MXenes demonstrate exceptional photothermal conversion efficiency and high thermal conductivity, positioning them as highly promising candidates for energy conversion and storage applications [104].

**Fig. 7.**
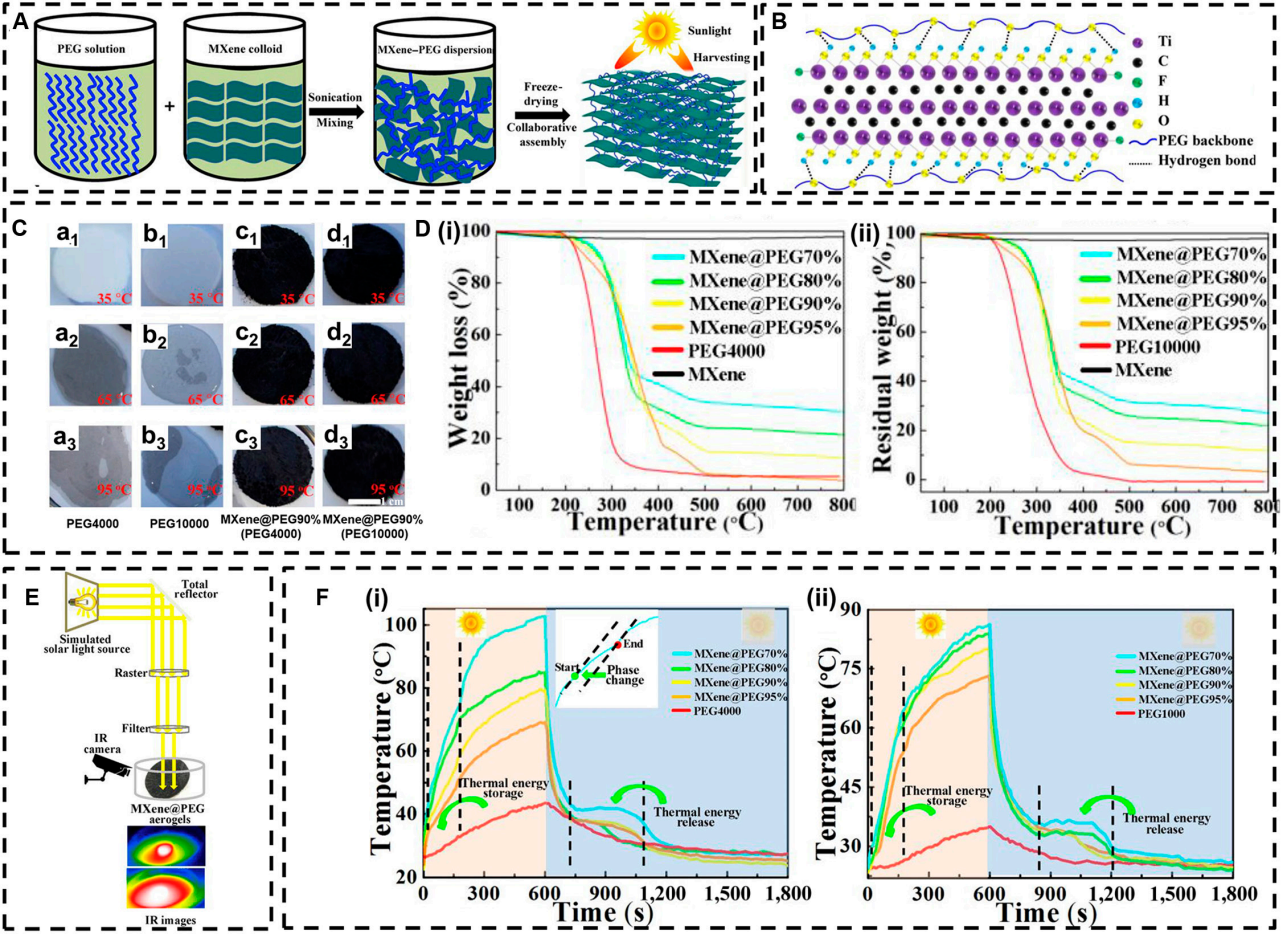
(A) Schematic diagram of MXene@PEG preparation. (B) The intensive interaction between Ti_3_C_2_T*_x_* nanosheets and polyethylene glycol (PEG) chains by the in situ hydrogen bonds. (C) Shape stability at different temperatures. (D) Mass loss curves of nanosheets with different MXene contents at different temperatures. (E) Experimental setup for the light-to-heat conversion of MXene@PEG aerogels. (F) Temperature evolution curves. Reproduced with permission from [105]. Copyright 2020, Elsevier Ltd. IR, infrared.

In a pioneering study, Lin et al. [105] measured the extinction coefficient of multilayer Ti_3_C_2_T*_x_* nanosheets at 808 nm, finding it to be higher than that of gold nanorods, reduced graphene oxide, and WS_2_ nanosheets, which underscores their excellent light absorption properties [106,107]. Subsequently, the researchers developed MXene@PEG aerogels by linking polyethylene glycol (PEG) chains to Ti_3_C_2_T*_x_* nanosheets through hydrogen bonding in solution, followed by freeze-drying (Fig. 7A and B). The resulting MXene@PEG aerogel exhibited a density of approximately 30 mg/cm^3^, confirming its lightweight nature. To evaluate the morphological stability of the MXene@PEG aerogel, the team compared it to control groups of PEG4000 and PEG10000, which were stored at different constant temperatures for 30 min to observe their morphological changes. As illustrated in Fig. 7C, all samples remained stable at 35 °C and gradually transitioned to a flowing liquid at 95 °C; however, the MXene@PEG aerogel retained its original shape due to the supportive 3D network of MXene nanosheets and the hydrogen bonding interactions between MXene and PEG. The maximum temperature at which the MXene@PEG aerogel remained stable was approximately 200 °C.

Additionally, MXene@PEG aerogels exhibited excellent thermal stability, with less than 3% weight loss occurring within the temperature range of 40 to 800 °C (Fig. 7D). This stability is attributed to the robust inorganic framework of the MXenes. The thermal stability significantly improved with increasing MXene content, primarily due to the inherent thermal stability of MXenes and the reduction in thermal conductivity provided by the aerogel’s porous structure. The researchers evaluated the photothermal storage efficiency of the MXene@PEG aerogels using the apparatus illustrated in Fig. 7E. They found that the photothermal storage efficiency of the MXene@PEG aerogel (utilizing PEG4000) reached an impressive 92.5% when the MXene content was 30% (see Fig. 7F). This study presents a novel scaffold for low-density and shape-stable photothermal carriers, establishing a foundation for the application of MXenes in solar energy technologies.

At present, the application of MXene-based materials in the field of phase change heat storage has garnered a lot of attention. These materials include a variety of composites, such as MXene/bacterial cellulose aerogel films [108] and PU/MXene composite materials [105,109]. Each of these configurations has demonstrated high performance as PCMs, indicating their capability for effective thermal energy storage. Their advantageous properties, including high thermal conductivity, excellent thermal stability, and efficient photothermal conversion, position them as key contributors to the advancement of thermal management technologies across various applications.

Phase change thermal storage represents a transformative approach to energy management, particularly in the context of renewable energy utilization and grid stability. By harnessing the latent heat associated with phase transitions, materials such as PCMs can efficiently store and release thermal energy, enabling systems to balance supply and demand. Future advancements in phase change thermal storage are likely to focus on enhancing the thermal conductivity and melting/solidification kinetics of PCMs through innovative material compositions, nanostructuring, and hybridization with conductive additives. These improvements aim to optimize charge and discharge rates, making phase change thermal storage systems more responsive to fluctuating energy supplies from renewable sources such as solar and wind. Moreover, the integration of phase change thermal storage with smart grid technologies and building energy management systems can facilitate dynamic adjustments of thermal loads, enhancing overall efficiency and reducing peak demand pressures. Research into encapsulation techniques also holds promise for increasing the longevity and reliability of PCMs in practical applications, thereby mitigating concerns about material degradation over time. As the push for sustainable energy solutions continues, phase change thermal storage is poised to play a pivotal role in advancing thermal management strategies for residential, commercial, and industrial applications, ultimately contributing to more resilient and efficient energy systems.

### EMI shielding

The popularity of wearable electronic devices is increasing due to their portability and multifunctionality, which has spurred heightened research in this field [110]. However, these miniaturized devices emit EMI that can adversely affect the performance of electronic systems [49]. In our information-driven era, exposure to electromagnetic radiation is nearly unavoidable. Prolonged exposure can lead to various health issues, including headaches and eye problems [111] and, in severe cases, even cancer [112]. For example, pregnant women who are exposed to prolonged electromagnetic radiation face risks to their babies’ brain development [113]. Furthermore, individuals with medical implants, such as hearing aids and pacemakers [114], may encounter health and safety risks from EMI that could compromise the functionality of these devices. Additionally, the extended use of integrated electronic components in wearable devices increases the risk of device fires due to heat accumulation and thermal failures. Therefore, there is an urgent need for EMI shielding films that also demonstrate effective fire prevention and heat dissipation properties to safeguard human life and health.

In 2016, Shahzad et al. [49] published the first report detailing the EMI performance of Ti_3_C_2_T*_x_*, paving the way to the application of MXene materials in EMI shielding. The Ti_3_C_2_T*_x_* film demonstrated an impressive EMI shielding effectiveness (SE) of 92 dB, surpassing that of other synthesized materials with comparable thicknesses at that time. This exceptional shielding performance can be attributed to the outstanding electrical conductivity of the Ti_3_C_2_T*_x_* film and the multiple internal reflections occurring within the film.

**Fig. 8.**
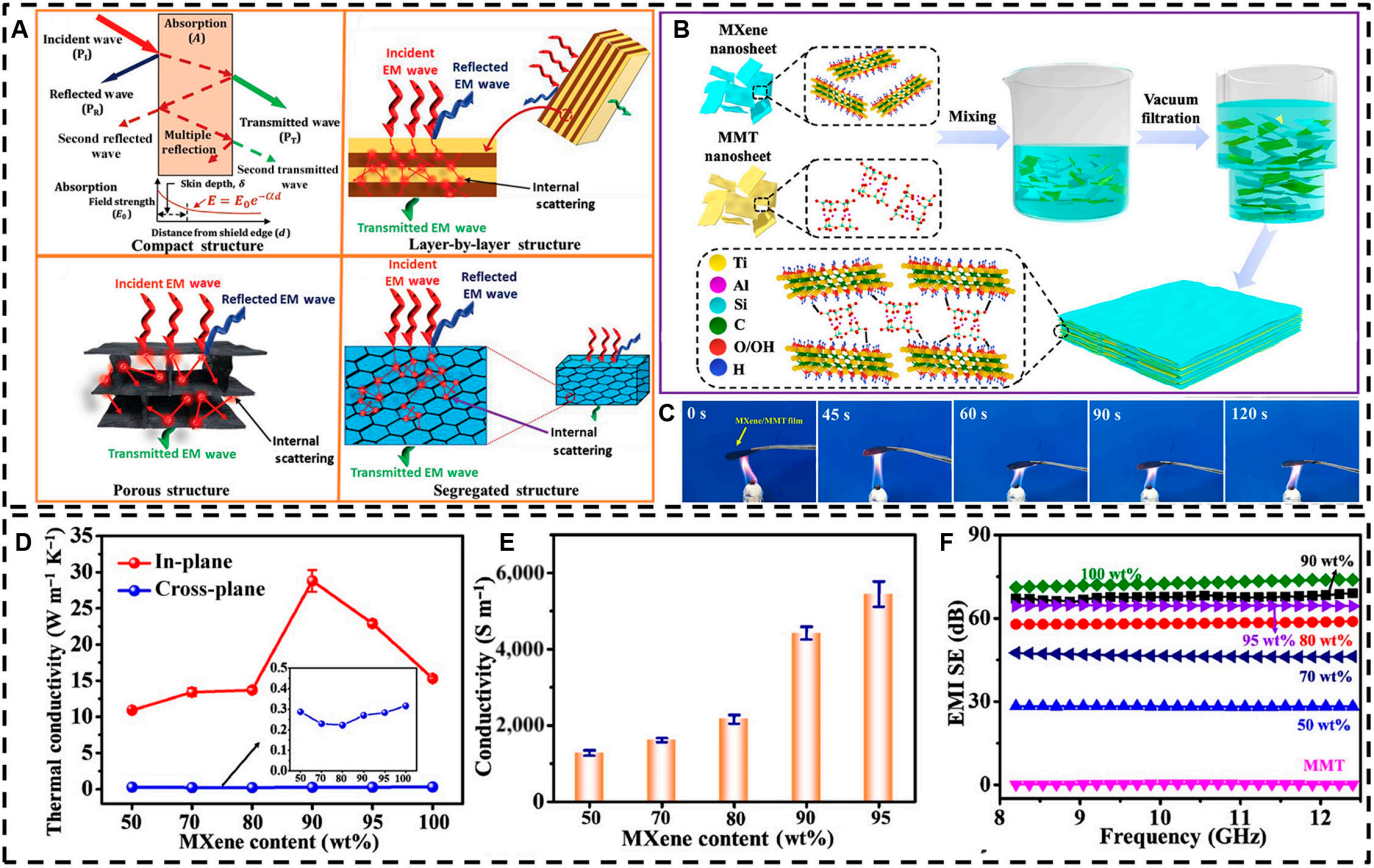
(A) The interaction between incident electromagnetic waves and materials with different structural forms. Reproduced with permission from [116]. Copyright 2020, John Wiley & Sons Inc. (B) Process diagram for preparing MXene/montmorillonite (MMT) composite films. (C) Fire resistance test of MXene/MMT composite films. (D) In-plane and cross-plane thermal conductivity of MXene and MXene/MMT composite films. (E) Electrical conductivity of MXene/MMT composite films. (F) EMI shielding effectiveness (SE) of pure MMT, MXene, and MXene/MMT composite films in the X-band. Reproduced with permission from [107]. Copyright 2020, American Chemical Society.

The excellent EMI shielding performance of MXenes is influenced not only by their inherent characteristics but also by external modifications [115]. Researchers have explored a variety of MXene composites and hybrids in multiple structural forms, including compact and laminate structures, layer-by-layer assemblies, porous foams and aerogels, and segregated architectures [116]. These efforts are all aimed at enhancing their intrinsic EMI shielding properties (see Fig. 8A). The Table summarizes the EMI SE and absolute shielding effectiveness (SSE/t) of MXene-based shielding materials across different structural configurations and compositions.

**Table.** EMI shielding performance of various structural forms based on MXenes

Type	Serial number	Composition	Thickness (mm)	SE (dB)	SSE/t (dB cm^2^·g^−1^)	Frequency (GHz)	Reference
Film	1	Ti_3_C_2_T*_x_*	0.045	92	8,554	8.2–12.4	[49]
	2	Ti_3_C_2_T*_x_*	0.001	48.4	130,000	8.2–12.4	[118]
	3	Ti_3_C_2_T*_x_*/PEDOT:PSS/PBO	0.026	ND	10,143.4	8.2–12.4	[119]
	4	Ti_3_C_2_T*_x_*@CNT/SA@Ti_3_C_2_T*_x_*	0.0012	61.3	234,000	8.2–12.4	[120]
	5	Ti_3_C_2_T*_x_*/PAN@-TiO_2_@PDA	0.028	32	4,085.92	8.2–12.4	[121]
	6	Ti_3_C_2_T*_x_*/AgNWs	0.12	54	ND	8.2–12.4	[122]
	7	PE@PET/Ti_3_C_2_T*_x_*	0.058	50.44	10,606	8.2–12.4	[123]
	8	Ti_3_C_2_T*_x_*/TiO_2_/graphene	0.00525	28	30,291	2–18	[124]
	9	Ti_3_C_2_T*_x_*/PEDOT:PSS	0.011	42.1	19,497.8	8.2–12.4	[125]
	10	CuNWs/Ti_3_C_2_T*_x_*/ANFs	0.043	46.67	9,120.6	8.2–12.4	[126]
	11	Ti_3_C_2_T*_x_*/CS	0.013	19.7	15,153.9	8.2–12.4	[127]
	12	Ti_3_C_2_T*_x_*/GO	0.003	22.6	2,053	2–18	[128]
	13	Ti_3_C_2_T*_x_*/ANF	0.012	44	18,847.6	8.2–12.4	[129]
Foam and aerogel	14	Ti_3_C_2_T*_x_*	0.06	70.6	53,030	8.2–12.4	[130]
	15	Ti_3_C_2_T*_x_*/rGO	7	83.3	3,119	8.2–12.4	[131]
	16	Ti_3_CNT*_x_*	1	42.3	76,909	8.2–12.4	[132]
	17	Ti_3_C_2_T_*x*_ aerogel/WPC	ND	71.3	362	8.2–12.4	[133]
	18	Ti_3_C_2_T*_x_*/CNF	2	44.5	1,844.6	8.2–12.4	[134]
Segregated	19	Ti_3_C_2_T*_x_*/PS	2	62	ND	8.2–12.4	[135]
	20	Ti_3_C_2_T*_x_*/p-PC	2	38.7	ND	8.2–12.4	[136]

SSE/t, absolute shielding effectiveness; ND, no data; AgNWs, silver nanowires; CuNWs, copper nanowires; GO, graphene oxide; PEDOT:PSS, poly(3,4-ethylene dioxythiophene)-polystyrene sulfonic acid; PBO, poly(*p*-phenylene benzobisoxazole); CNT/SA, carbon nanotube/sodium alginate; PAN, polyacrylonitrile; PDN, polydopamine; PE, polyethylene; PET, polyethylene terephthalate; ANFs, aramid nanofibers; CS, chitosan; WPC, wood-derived porous carbon; CNF, carbon nanofibers; PS, polystyrene; p-PC, positively charged polycarbonate

Moreover, the EMI shielding capacity of MXenes is also related to their thickness. Yury Gogotsi [109] and colleagues systematically examined the EMI shielding properties of Ti_3_C_2_T*_x_* assembly films, ranging from single-layer to multilayer thicknesses. Their theoretical model elucidated the shielding mechanisms occurring below the skin depth, where multiple reflections become important alongside surface reflection and substantial absorption of electromagnetic radiation. These findings underscore that EMI shielding in MXene materials is influenced by various factors, necessitating the careful selection of appropriate MXenes tailored to specific application requirements.

Recently, Li et al. [107] reported on a multifunctional fire-resistant EMI shielding film (Fig. 8B). Their study demonstrated that incorporating montmorillonite (MMT) can enhance the antioxidative properties of MXenes, resulting in a composite film that exhibits exceptional fire resistance (Fig. 8C) and remarkable thermal conductivity (Fig. 8D). Generally, as the conductivity of a shielding material improves, its EMI performance also enhances. When the MXene content in the composite membrane reached 90 wt%, the conductivity attained 4,420 S/m (Fig. 8E), indicating a strong EMI shielding effect (Fig. 8F). Moreover, it is worth noting that the EMI SE of all MXene/MMT composite films met commercial standards (>20 dB). Impressively, even after being subjected to flames for 30 s, the EMI SE of the MXene/MMT composite film remained above 60 dB. Additionally, the composite demonstrated outstanding Joule heating performance. These multifunctional films hold important promise for applications in fire prevention, in heat dissipation and insulation, and as EMI shielding devices.

In summary, MXenes have emerged as highly effective materials for EMI shielding, owing to their exceptional intrinsic properties, which include high conductivity, lightweight characteristics, and ease of processing. The tunable surface chemistry of MXenes further enhances their utility by enabling the design of composite materials with controllable structures, thereby broadening their applicability across various fields.

## Summary and Perspectives

In conclusion, MXenes hold substantial promise for development in thermal applications, attributable to their superior electrical and thermal conductivity, exceptional photothermal conversion efficiency, and effective light absorption. Their inherent flexibility, EMI shielding capabilities, and efficient photothermal performance render them particularly well suited for modern wearable devices. Additionally, MXenes demonstrate great potential in biomedical applications, contributing importantly to human health and safety. The outstanding thermal properties of MXene materials will facilitate the effective use of thermal energy, thereby accelerating advancements in personal thermal management, electronic device heat dissipation, and military applications, all while addressing energy depletion concerns.

Despite the progress made in MXene research for thermal applications, several unresolved challenges remain that warrant ongoing scientific exploration and practical application:

• Exploring diverse MXene types: Research has predominantly focused on Ti_3_C_2_T*_x_* regarding microwave absorption and thermal conductivity, with limited exploration of other MXene types. Comprehensive investigations into alternative MXene materials are needed. Furthermore, while numerous individual research on the microwave absorption and thermal conductivity of MXenes and their composites, studies examining multifunctional integrated composite materials are sparse. To align with the future demands of electronic devices, it is important to advance the development of multifunctional materials. This advancement necessitates a deeper understanding of the mechanisms governing both microwave absorption and thermal conductivity.
• Suitable synthesis methods: Theoretical calculations indicate that MXene materials possess high thermal conductivity. However, due to various factors in the preparation process, the actual thermal conductivity of macroscopic MXene materials, such as thin films, is obviously lower than the theoretical values. Therefore, selecting an appropriate preparation method is crucial for obtaining MXene materials with superior thermal management performance. Moreover, as the demand for these versatile materials continues to grow across a range of applications—from energy storage to biomedical technologies—it is essential to explore sustainable synthesis methods for MXenes. Future research should prioritize the functional customization of MXenes and their composites, focusing on eco-friendly and cost-effective approaches that minimize the use of hazardous chemicals and reduce waste. Additionally, increasing the yield through efficient mass-production techniques (e.g., roll coating and spraying) can make the production of MXene materials more economical. The optimization of the manufacturing process is not only related to the cost-effectiveness of MXenes but also plays a vital role in facilitating larger-scale applications.
• Understanding the conversion mechanism: Although the Ti_3_C_2_T*_x_* MXene exhibits high photothermal conversion efficiency, its underlying conversion mechanism remains inadequately understood. Gaining insight into the mechanism of photothermal conversion is important for enhancing the efficiency and applicability of materials utilized in solar energy harvesting and thermal management applications. Furthermore, the structure of MXenes can be engineered based on this mechanism, and preparation methods can be developed to achieve the desired properties. Future research should focus on elucidating the intricate process by which MXene materials absorb light and convert it into heat. A comprehensive understanding of these mechanisms will also facilitate the development of hybrid systems that integrate photothermal and thermoelectric conversion, thereby maximizing the utilization of solar and electrical energy while improving overall system performance. As the emphasis on renewable energy continues to intensify, a thorough understanding of the photothermal conversion process is essential for advancing innovative solutions that support the transition to a more sustainable energy landscape.
• Multifunctional composite development: Currently, most reports regarding the thermal applications of MXenes indicate that the mechanical properties of MXene-based materials are inadequate, which limits their practical use in everyday life. By incorporating MXenes with other heat-conducting materials, including polymers, ceramics, and carbon nanotubes, MXene composites can achieve a balance between thermal conductivity and mechanical stability. For example, MXenes can be integrated with polymer materials to produce highly thermally conductive and flexible composites that are suitable for the thermal management of flexible electronic devices. The combination of MXenes with ceramic materials enhances high-temperature resistance while minimizing discrepancies in thermal expansion coefficients, making them ideal for heat dissipation devices in harsh environments. These multifunctional composites not only expand the application areas of MXenes but also address various thermal management needs, providing more competitive solutions for thermal management.
• Thermoelectric devices and energy recovery: MXenes exhibit high electrical conductivity and controllable thermal conductivity, presenting new opportunities for thermoelectric materials and energy recovery applications. These thermoelectric materials can convert heat energy into electricity, demonstrating great potential in waste heat recovery and mobile power generation. The Seebeck coefficient and conductivity of MXenes can be enhanced by modifying their electronic structure and optimizing the thermal conductivity pathways, thereby improving thermoelectric conversion efficiency. Furthermore, investigating the stability and lifespan of MXene composites in thermoelectric devices will yield innovative solutions for waste heat recovery and energy recovery applications. In the future, MXene-based thermoelectric materials may play a crucial role in energy conservation and emission reduction within industrial production and electronic equipment, ultimately contributing to enhanced energy efficiency.

Since the discovery of MXenes 13 years ago, important advancements have been made in their preparation, performance, and potential applications, particularly in thermal contexts. The thermal properties of MXene materials continue to improve alongside ongoing technological advancements in materials science. As research methodologies and systems for studying MXenes evolve, it is anticipated that MXene materials will become increasingly integrated into daily life.
